# The Malaria Box: a catalyst for drug discovery

**DOI:** 10.1186/1475-2875-11-S1-P136

**Published:** 2012-11-09

**Authors:** Jeremy Burrows, Paul Kowalczyk, Simon McDonald, Thomas Spangenberg, Timothy Wells, Paul Willis

**Affiliations:** 1 Medicines for Malaria Venture, route de Pré-Bois 20, PO Box 1826, Geneva, Switzerland; 2 SCYNEXIS Inc. 3501 Tricenter Boulevard Durham, NC, USA

## 

The discovery of new chemotypes to feed the pipeline of antimalarial drugs remains a constant challenge, particularly in light of emerging resistance to current therapies. Recently, phenotypic screenings have been successfully used for antimalarial hit generation where the biological target(s) may often not be clearly identified. To catalyse malaria research by both filling the pipeline and having a better understanding of ligand-target relationships, a unique screening tool has been elaborated: the Malaria Box.

The Malaria Box is a set composed of 400 commercially available chemical entities derived from a selection of more than 20,000 hits from the screening of corporate and academic libraries [1-3]. The originality of the Malaria Box relies in its composition of 200 lead-like and 200 probe-like compounds that have confirmed activity on blood-staged Plasmodium falciparum and that have been assessed for cytotoxicity. Lead-like compounds commensurate with oral absorption and the presence of known toxicophores has been reviewed. Conversely probe-like compounds are intended to represent the broadest cross-section of structural diversity.

Significantly, the scope of the Malaria Box goes beyond the Malaria field as active compounds may have utility in other parasitic or neglected diseases. It is well-documented that artemisinin was initially discovered from helminth research and is currently a gold standard drug against Malaria. Also, the presence of orthologues of various molecular targets may lead to new therapeutic applications in orphan diseases or for example oncology. Ultimately, the data collection resulting from the Malaria Box would enable the community to better understand similarities and differences between parasite diseases or orphan diseases by mining data sets that were previously considered separately [4].

Herein we disclose the selection process applied to assemble the Malaria Box as well a preliminary results.
